# Dynamics of BMP and Hes1/Hairy1 signaling in the dorsal neural tube underlies the transition from neural crest to definitive roof plate

**DOI:** 10.1186/s12915-016-0245-6

**Published:** 2016-03-24

**Authors:** Erez Nitzan, Oshri Avraham, Nitza Kahane, Shai Ofek, Deepak Kumar, Chaya Kalcheim

**Affiliations:** Department of Medical Neurobiology, IMRIC and ELSC, Hebrew University of Jerusalem-Hadassah Medical School, Jerusalem, 9112102, PO Box 12272,, Israel; Present Address: Department of Molecular Cell Biology, Weizmann Institute of Science, Rehovot, Israel; Present address: Department of Genetics, Washington University, St. Louis, MO USA

**Keywords:** Avian embryo, BMP, Epithelial-to-mesenchymal transition, Foxd3, Hairy1, HES1, Dorsal interneurons, Dorso-ventral patterning, Neural tube

## Abstract

**Background:**

The dorsal midline region of the neural tube that results from closure of the neural folds is generally termed the roof plate (RP). However, this domain is highly dynamic and complex, and is first transiently inhabited by prospective neural crest (NC) cells that sequentially emigrate from the neuroepithelium. It only later becomes the definitive RP, the dorsal midline cells of the spinal cord. We previously showed that at the trunk level of the axis, prospective RP progenitors originate ventral to the premigratory NC and progressively reach the dorsal midline following NC emigration. However, the molecular mechanisms underlying the end of NC production and formation of the definitive RP remain virtually unknown.

**Results:**

Based on distinctive cellular and molecular traits, we have defined an initial NC and a subsequent RP stage, allowing us to investigate the mechanisms responsible for the transition between the two phases.

We demonstrate that in spite of the constant production of BMP4 in the dorsal tube at both stages, RP progenitors only transiently respond to the ligand and lose competence shortly before they arrive at their final location. In addition, exposure of dorsal tube cells at the NC stage to high levels of BMP signaling induces premature RP traits, such as *Hes1*/*Hairy1*, while concomitantly inhibiting NC production. Reciprocally, early inhibition of BMP signaling prevents *Hairy1* mRNA expression at the RP stage altogether, suggesting that BMP is both necessary and sufficient for the development of this RP-specific trait.

Furthermore, when Hes1/Hairy1 is misexpressed at the NC stage, it inhibits BMP signaling and downregulates *BMPR1A/Alk3* mRNA expression, transcription of BMP targets such as *Foxd3*, cell-cycle progression, and NC emigration. Reciprocally, Foxd3 inhibits *Hairy1*, suggesting that repressive cross-interactions at the level of, and downstream from, BMP ensure the temporal separation between both lineages.

**Conclusions:**

Together, our data suggest that BMP signaling is important both for NC and RP formation. Given that these two structures develop sequentially, we speculate that the longer exposure of RP progenitors to BMP compared with that of premigratory NC cells may be translated into a higher signaling level in the former. This induces changes in responsiveness to BMP, most likely by downregulating the expression of Alk3 receptors and, consequently, of BMP-dependent downstream transcription factors, which exhibit spatial complementary expression patterns and mutually repress each other to generate alternative fates. This molecular dynamic is likely to account for the transition between the NC and definitive RP stages and thus be responsible for the segregation between central and peripheral lineages during neural development.

**Electronic supplementary material:**

The online version of this article (doi:10.1186/s12915-016-0245-6) contains supplementary material, which is available to authorized users.

## Background

The fundamental decision whether to become peripheral nervous system (PNS) or central nervous system (CNS) takes place in the dorsal domain of the developing neural tube (NT). In this area three main populations of cells are sequentially generated. First to appear are various populations of neural crest (NC) cells, consisting of precursors of most neurons and all glia of the PNS as well as melanocytes, endocrine derivatives, etc. [1–3]. Second to appear are roof plate (RP) cells, which constitute the definitive dorsal midline of the CNS, and third are dorsal spinal interneurons, whose specification and differentiation are controlled by dorsal NT-derived signals [4–8].

Only selected aspects of dorsal NT development have been experimentally addressed, and many essential questions remain unanswered. For instance, the mechanisms responsible for the ordered transition between NC and RP are largely unknown. Although prospective premigratory NC and RP cells are not distinguishable at the early stages either morphologically or by molecular means, NC progenitors proliferate both before as well as after delamination, and in the trunk they synchronously exit the epithelium at the S phase of the cell cycle [9]. In contrast, RP cells become post-mitotic [10] and adopt their characteristic morphology, constituting the definitive dorsal domain of the spinal cord.

Single cell lineage tracing suggested that NC and NT progenitors derive from a common founder cell [11]. Nonetheless, these studies did not identify the precise CNS cell types involved (i.e., RP, specific interneurons, or both), neither did they determine the exact stage(s) in which this putative common founder cell prevails or segregates. Hence, questions remain, such as where in the dorsal NT do RP cells originate and when precisely do they segregate from the NC lineage? Using spatiotemporally controlled lineage analysis, our previous findings revealed that the dorsal NT is sequentially transited by distinct cell populations that exit the NT to populate NC derivatives [12, 13]. In one of these studies it was found that RP progenitors originate ventral to the premigratory NC and relocate ventro-dorsally to reach their final position in the dorsal midline of the NT upon completion of NC exit [12]. Furthermore, tracing the dynamics of the NC marker Foxd3 using a specific reporter revealed that NC and RP progenitors are initially part of the Foxd3 lineage, yet RP precursors downregulate its expression while relocating into the dorsal midline; this occurs when some NC progenitors still reside within the neuroepithelium [13]. Hence, it is most likely that RP precursors share a common lineage with NC only at an early stage of dorsal NT development. This domain is, therefore, a dynamic area in which progressive NC emigration takes place until replacement by the definitive RP, thus resulting in the separation between CNS and PNS [4, 12].

The signaling activity of bone morphogenetic proteins (BMPs) is essential for NC induction, epithelial-to-mesenchymal transitions (EMTs), and specification and patterning of dorsal interneurons [6, 8, 14–19]. But whether BMP signaling is necessary for RP development and what distinguishes between RP and NC in terms of BMP activity remains to be addressed. The transcription factors Lmx1a and 1b are highly expressed in the dorsal NT and differentiated RP cells in the chick developing spinal cord. They were reported to act downstream to BMP and to affect expression of RP markers and interneuron development [20, 21]. However, given that Lmx genes in turn stimulate expression of both BMP and Wnt [20, 21], the possibility exists that BMP acts on RP and/or interneuron production independently of Lmx or that additional downstream factors are necessary.

In the present study, we characterize the nascent RP as an epithelial group of cells exhibiting apico-basal polarity and apically localized cilia, little or no cell proliferation, expression and activity of the basic helix-loop-helix (bHLH) transcriptional repressor Hes1/Hairy1 [22, 23], and transcription of the ciliary protein *Foxj1* [24]. This contrasts with the proliferative NC stage in which dorsal NT cells are devoid of, or discontinuously express, N-cadherin and laminin while transcribing typical NC markers such as *Foxd3*, *Snail2,* and *Sox9* [4, 12, 13]. Furthermore, we show that despite the constant production of BMP4 in the dorsal NT, RP progenitors only initially respond to the ligand and lose competence during their ventro-dorsal relocation towards the midline of the NT, with a concomitant downregulation of *BMPR1A/Alk3*. Because both NC and RP progenitors are localized within a domain of high BMP activity [18], we hypothesized that RP progenitors are also sensitive to BMP signaling. Indeed, constitutive activation of the BMP pathway resulted in premature transcription of the RP marker *Hairy1*. Notably, this was associated with an inhibition of NC production. Furthermore, early misexpression of Hes1/Hairy1 at the NC stage inhibited BMP signaling while downregulating expression of the *Alk3* receptor, transcription of BMP targets such as *Foxd3*, cell-cycle progression, and NC emigration. Conversely, Foxd3 inhibited *Hairy1* altogether, suggesting that repressive cross-interactions at the level of and downstream of BMP ensure the temporal separation between the two lineages.

## Results

### Cellular characterization of the dorsal NT at NC and RP stages

**Fig. 1:**
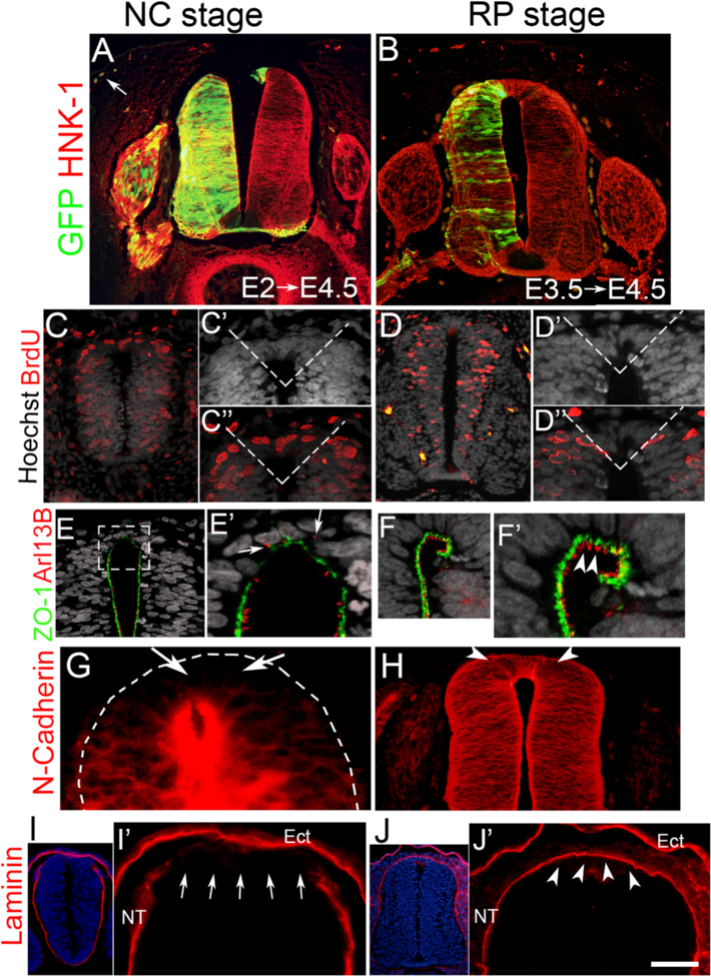
Differential cellular characteristics of the neural tube at neural crest (*NC*) and roof plate (*RP*) stages. **a**, **b** Transverse sections of the flank region of E4.5 avian embryos whose hemi-neural tubes (*NTs*) were electroporated with a control GFP plasmid at E2 (**a**) or E3.5 (**b**). Note the contribution of labeled cells to NC derivatives including melanocytes (*arrow* in a) following early but not late stage electroporations. **c**, **d** Bromodeoxyuridine (*BrdU*) incorporation following a 1-h pulse at NC (E2–E2.5, c) or RP (E3.5, d) stages. *Dashed lines* in insets mark the dorsal NT domain that was quantified (see text for details). Note the presence of the BrdU+ nuclei (Red) in **c**–**c”** but not in the equivalent dashed area in **d**–**d”**. Nuclei were visualized with Hoechst. **e**–**j** Antibody staining for epithelial (ZO-1, N-cadherin, laminin) or ciliary (Arl13b) markers. *Arrows* point to disorganized cilia (**e’**), the absence of N-cadherin in the dorsal NT compared to more ventral regions (**g**), and an incomplete laminin-containing basal lamina (**i**, **i’**) at the NC stage. In contrast, note the apically oriented cilia (**f**), positive N-cadherin immunostaining (**h**), and continuous laminin expression (**j**) in the dorsal NT at the RP stage (*arrowheads* in **f**, **h**, and **j’**). *Ect* ectoderm. Bar in **a**, **b**, **d**, **h**, **j** = 80 μM; **c** = 50 μM; **c’**, **d’**, **e** = 30 μM; **f**, **g**, **i’** = 40 μM; **e’** =15 μM; **j** = 240 μM; **i** =140 μM

To begin understanding the mechanisms underlying the transition from the NC to the RP stage, we first characterized differences in cellular behavior between the two phases. Following electroporation of a GFP-encoding DNA into the flank-level NT at embryonic day 2 (E2) (Hamburger and Hamilton stages HH12–14, 17–20 somite stage [ss]), we observed labeled NC derivatives in the periphery at E4.5 (Fig. 1a). GFP+ cells were found in characteristic positions, such as the sympathetic ganglia, along nerves as Schwann cells, in the dorsal root ganglion (DRG), and in the dermis corresponding to melanocytes (see also [12]). In contrast, electroporation of GFP at E3.5 (40 ss, HH19–20) followed by fixation at E4.5 did not produce any labeled cells outside the neuroepithelium (Fig. 1b), in agreement with our previous results [12]. This suggests that by E3.5 at flank levels of the axis, all NC progenitors have already left the dorsal neuroepithelium, which is now occupied by definitive RP cells. Hence, we refer to the period between E2 and E3.5 in which the dorsal NT produces emigrating cells as the “NC stage” and to the period starting from E3.5 onward as the “RP stage.”

We next characterized the dynamics of cell proliferation in the dorsal NT, because the transition from the G1 to the S phase of the cell cycle was found to be critical for NC delamination [9]. To this end, an area containing 8–10 nuclei per hemi-NT was considered in each section (dashed lines in Fig. 1c, d). These cells are included within the expression domain of *Foxd3* and *MafB* at the NC and RP stages, respectively. At the NC stage, 45.9 ± 4.0 % of the cells in the dorsal NT had incorporated BrdU following a 1-h pulse, consistent with previous findings [17]. At more advanced stages of NC delamination, the proportion of BrdU+ nuclei decreased to 26.4 ± 3.4 %, and by E3.5 only 4.8 ± 0.5 % of the nuclei were BrdU+ (N = 5 embryos/stage, Fig. 1c, d). Thus, the shift from the NC stage to the RP stage involves cell-cycle exit [10], which is associated with the end of NC emigration.

Next, we examined the expression of several proteins that characterize embryonic epithelia. At the NC stage, the apical epithelial markers ZO-1 and N-cadherin were discontinuous or virtually absent in the dorsal NT, respectively (Fig. 1e, g) [25]. In addition, Arl13b-positive cilia [26] were randomly distributed in the dorsal NT rather than pointing apically into the NT lumen as observed at more ventral regions of the neuroepithelium (Fig. 1e, e’). Furthermore, the laminin-expressing basement membrane at the basal side of the dorsal NT was discontinuous (Fig. 1i, i’, see also [27, 28]). In contrast, a close association between ZO-1 and Arl13b in the apical side of the NT was apparent at the RP stage (Fig. 1f, f’), N-cadherin was re-expressed (Fig. 1h), and the laminin-positive basal lamina was uninterrupted (Fig. 1j, j’). These observations suggest that the nascent RP regains epithelial characteristics such as apico-basal polarity, in spite of these being disrupted during the period of NC delamination.

### Shared and differential transcriptional gene patterns in the dorsal NT at NC and RP stages

To elucidate the mechanisms leading to the transition between NC and RP stages, it would be useful to identify and functionally characterize genes whose expression is restricted to either stage. To this end, we examined expression patterns of candidate dorsal NT markers.

The Id family of transcriptional regulators encodes four HLH proteins that lack a basic DNA-binding domain, and function in a dominant negative manner by binding and sequestering bHLH transcription factors into inactive heterodimers [29, 30]. Id2 was reported to regulate NC specification, and to maintain the balance between cell differentiation and proliferation [31]. Ids are also known effectors of BMP signaling [32–34]. *Id2* and *Id3* were expressed in the dorsal NT at the NC stage, but not in the definitive RP, and at the RP stage *Id2/3* only appeared ventral to the RP, likely in dorsal interneuron progenitors (Additional file 1: Figure S1A–D). In contrast, no *Id1* or *Id4* were apparent in the dorsal NT (not shown). Leukocyte tyrosine kinase (*LTK*) is expressed in zebrafish NC cells, and in particular in iridophores, where it was shown to be critical for their development [35]. *LTK* is also expressed in migrating avian cranial NC [36]. In the trunk, *LTK* was transcribed in the dorsal NT at the NC but not RP stage (Additional file 1: Figure S1E, F). Additionally, transcription of *Foxd3*, *Sox9*, and *Snail2* was restricted to the neural progenitors of the NC and absent from the definitive RP (Additional file 1: Figure S1G, H and see [12]).

**Fig. 2:**
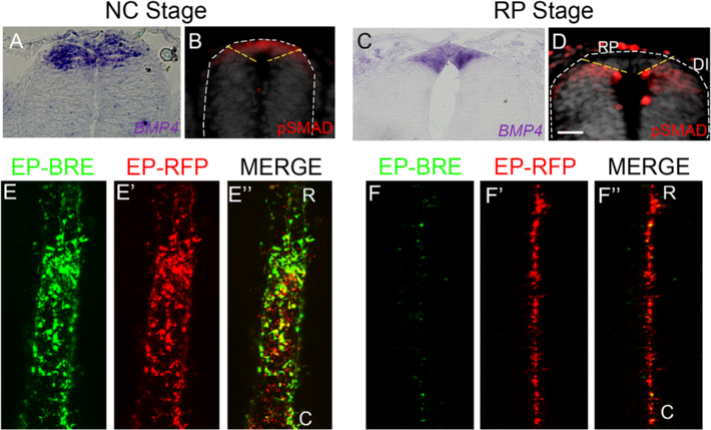
The roof plate (*RP*) loses responsiveness to BMP signaling. **a**, **c** In situ hybridization for *Bmp4* at the neural crest (*NC*) and RP stages. **b**, **d** Antibody staining for pSMAD1,5,8. Note positive signaling in the dorsal neural tube (*NT*) at the NC stage (delimited by *dotted yellow lines*, see “Methods” for definition). In contrast, the definitive RP (dorsal to the dotted yellow lines, see “Methods” for definition) lacks a pSMAD signal yet the domain ventral to the yellow lines containing dorsal interneurons is positive. **e**, **f** Dorsal views of whole mounted embryo fragments following dorsally directed electroporations (*EP*) of BMP-responsive element (*BRE*)-GFP along with control RFP to monitor electroporation efficiency. Note positive and negative BMP reporter signaling at NC and RP stages, respectively. Embryos were electroporated at either E2 (**e**–**e”**) or E3 (**f**–**f”**) and analyzed 16 h later. *White dashed lines* delineate the NT in **b** and **d**. *C* caudal, *DI* dorsal interneurons, *R* rostral. Bar in **a** = 40 μM; **b**, **d** = 80 μM; **c** = 50 μM

The transcription factor Foxj1 has been implicated in the formation of motile cilia. In the floor plate (FP), Foxj1 alters responsiveness of these ventral midline cells to Sonic hedgehog (Shh), prompting them to become refractory [24]. Evidence also points to its regulation by BMP signaling [37]. *Foxj1* mRNA was only evident at the RP but not the NC stage, despite being transcribed continuously in the FP (Additional file 1: Figure S1I, J). *Hairy1*, a bHLH transcriptional repressor of the Hairy/Hes family, revealed a similar profile of expression to that of *Foxj1* (Additional file 1: Figure S1K, L). Thus, while *Id1/2*, *LTK*, *Foxd3*, *Snail2*, and *Sox9* are differential markers for the early NC stage, *Foxj1* and *Hairy1* differentially map to the RP stage. Notably, *Bmp4*, *Gdf7*, *Wnt1*, *MafB*, and *Lmx1a/1b* are continuously expressed throughout NT development, even if classically defined as RP markers (Fig. 2a, c; Additional file 1: Figure S1M–R; and see [4, 14, 20, 21]). Taken together, the above cellular and molecular traits differentially define two discrete phases in dorsal NT development: NC and RP.

### BMP signaling is transient in the dorsal midline of the NT

#### The definitive RP cells are refractory to BMP signaling

Next, we began addressing the mechanisms responsible for the transition between these stages. During patterning of the nervous system, the dorsal NT produces and secretes proteins of the BMP family that at early stages control the emigration of NC [16, 17, 25, 38] and later induce, in a non-cell autonomous manner, the specification and differentiation of spinal interneurons [39, 40]. These activities of BMP prompted us to examine its involvement in the transition between NC and RP stages during dorsal NT development.

In agreement with previous findings [4, 12], we observed that expression of *Bmp4* transcripts was evident in the dorsal NT at both NC and RP stages (Fig. 2a, c). BMP activity was evidenced with anti-Phospho-Smad 1-5-8 (pSMAD) [41]. In contrast to *Bmp4* transcription, pSMAD staining was positive in the dorsal NT only at the NC stage (Fig. 2b). At the RP stage, only dorsal interneurons, located ventral to the definitive RP, were pSMAD+, while the midline RP domain was negative (Fig. 2d).

To further examine this stage-dependent difference in BMP sensitivity, we took advantage of a genetic reporter for BMP activity, consisting of a BMP responsive element (BRE) that drives expression of GFP [8, 18]. Focal electroporations of BRE-GFP together with RFP to control for transfection efficiency were directed to the dorsal NT at E2 (17–20 ss) or E3 (35 ss). Dorsal views of transfected neural primordia revealed many RFP+ cells at the NC stage exhibiting a BRE:GFP signal (Fig. 2e–e”), whereas only a few RFP+ cells approaching the RP stage were BRE:GFP+ (Fig. 2f–f”) when examined 16 h after electroporation. Hence, while both NC and RP cells produce and secrete BMP proteins, only progenitors at the NC stage are responsive. The definitive RP loses competence to respond to the BMP ligand, while continuing to provide BMP to the ventrally localized interneurons and/or their progenitors.

#### Prospective RP cells are initially responsive to BMP

**Fig. 3:**
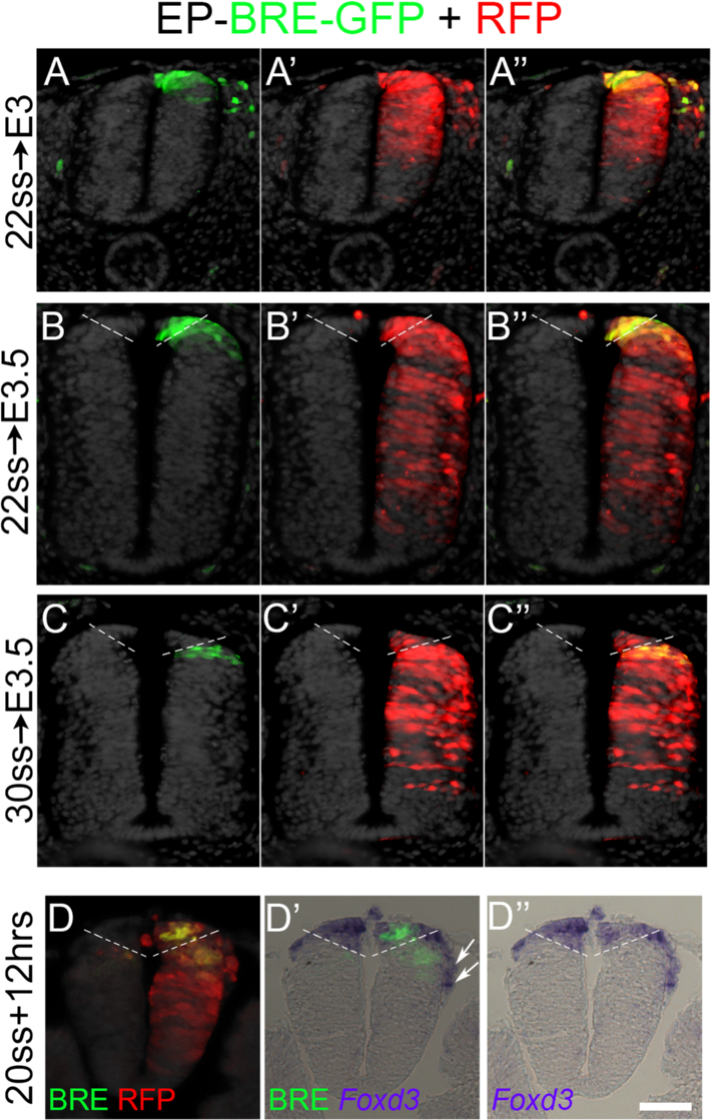
Dynamics of BMP responsive element (*BRE*) in the dorsal neural tube (NT). **a**–**c** Hemi-NT electoporations of BRE-GFP along with control RFP to trace electroporation efficiency. Embryos were electroporated and analyzed at designated times. Note presence of positive BRE-GFP signal in RFP+ cells in the early electroporations (**a**–**b”**). When electroporated later (**c**–**c”**), the BRE-GFP signal is absent in the definitive roof plate (RP) but positive more ventrally. *Dashed lines* in **b** and **c** delimit the definitive RP (see “Methods”). **d**–**d”** In situ hybridization for *Foxd3* transcripts, showing co-localization with the BRE-GFP signal in the dorsal NT. *Dashed lines* mark the ventral limit of the *Foxd3*-positive domain. Note also the presence of *Foxd3+* neural crest cells migrating dorso-ventrally outside the neuroepithelium (*arrows*). In addition, the BRE-GFP signal also extends further ventrally into a *Foxd3*-negative region. Bar for **a**–**a”** = 50 μM; **b**–**d”** = 40 μM

In a previous study [12] we demonstrated that preceding the onset of NC delamination, RP progenitors cells are located ventral to the presumptive NC pool, and that they relocate dorsally upon NC emigration. In addition, we found that, initially, prospective RP progenitors are part of the Foxd3+ lineage, but downregulate its expression during dorsal relocation [13]. We then hypothesized that RP progenitors are initially responsive to BMP signaling, given that all dorsal NT cells appear to be sensitive to BMP signaling at the NC stage (Fig. 2b, e). To test this notion, we transfected BRE-GFP to the flank of 22 ss (HH14)-stage embryos corresponding to the early NC stage. Embryos were re-incubated for an additional 24 or 36 h until reaching the RP stage. As predicted, the midline RP domain was BRE-GFP+ (Fig. 3a–a”, b–b”). Because the RP is already insensitive to BMP by the time of fixation, (e.g., BRE-GFP−, see Fig. 2), the GFP signal observed in the dorsal NT at E3–E3.5 must result from an accumulation of the GFP protein following the early electroporation. Because GFP has a half-life of about 48 h, it is possible to trace cells after the BMP signal has been turned off [42].

To control for this dynamic behavior, we performed similar electroporations a few hours later, in embryos aged 28–30 ss (HH16–17), still well within the NC stage [12, 43]. Under these conditions, the RP was largely negative for the BRE-GFP signal (Fig. 3c–c”). In this time frame, prospective RP cells are either unable to accumulate enough GFP before losing their capacity to respond to BMP, or they have already lost BMP sensitivity. These results reveal a similar temporal dynamic to that of RP progenitors, which only transiently exhibited Foxd3 reporter activity [13]. For this reason, we analyzed embryos that were injected at an early stage with BRE-GFP and then in situ hybridized for *Foxd3.* The *Foxd3*+ signal was included within the dorsal domain of BRE-GFP+ expression, encompassing at this stage both NC and RP precursors [13]. In addition, the BRE-GFP signal extended further ventrally into a *Foxd3*− region, likely comprising dorsal interneuron progenitors (Fig. 3d–d”). Together, these results suggest that prospective RP progenitors initially respond to BMP and then lose sensitivity upon relocation to their definitive dorsal midline position.

Because BMP ligands are continuously produced by dorsal NT cells, we hypothesized that the development of RP insensitivity to BMP may be accounted for by a timely downregulation of BMP receptors. In situ hybridization revealed that while expression of *Bmpr1b* (*Alk6*) transcripts is apparent in the dorsal NT at both NC and RP stages, that of *Bmpr1a* (*Alk3*) is positive at the NC stage but downregulated at the RP stage (Additional file 2: Figure S2). This indicates that the downregulation of specific BMP receptors is part of a mechanism responsible for the loss of competence of nascent RP cells to respond to BMP.

### The dynamics of BMP signaling vis-à-vis RP progenitors

Our results suggest that RP progenitors will respond to BMP signaling until they arrive at their dorsal midline position. Notably, because of their initial ventral localization with respect to the NC and their prolonged ventro-dorsal relocation, RP progenitors are likely subjected to BMP for longer than the NC cells, which progressively exit the NT. It was previously suggested that extended exposure to morphogens such as BMP and Shh is equivalent to generating a higher level of signaling required for the specification of distinct dorsal [18] and ventral [44] neuronal subsets, respectively. In such a case, exposing early neural progenitors to high BMP signaling should prematurely induce RP at the expense of NC. To test this possibility, a constitutively active (ca) version of Alk3 was used. In all cases (N = 6), when electroporated into hemi-NTs at 18–20 ss and fixed a day later, an ectopic pSMAD signal was detected in transfected cells. This contrasted with an observed pSMAD expression restricted to the dorsal domain under control conditions (N = 4) (Additional file 3: Figure S3).

**Fig. 4:**
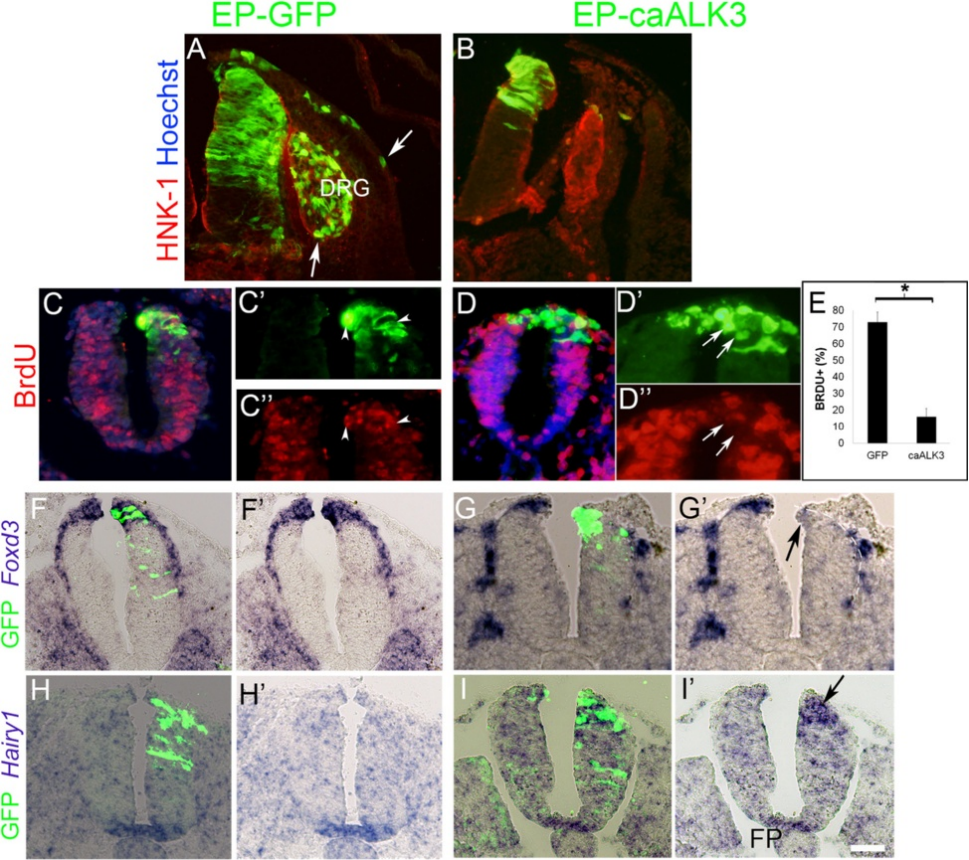
Early exposure to constitutively active (*ca*)Alk3 inhibits neural crest (*NC*) traits and stimulates premature transcription of Hairy1. **a**, **b** Transverse sections of control GFP (**a**) showing labeled cells that colonized the dorsal root ganglion (*DRG*) primordium as well as ectoderm (*arrows*). In contrast, no significant NC cell emigration is evident in caAlk3-GFP transfected neural tubes (NTs) (**b**). The NC marker HNK1 is in *red*. **c**, **d** Transverse sections following an extended 2-h pulse with BrdU. Embryos were sacrificed 16 h after a dorsally directed electroporation of either control GFP (**c–c”**) or caAlk3 (**d–d”**). *Arrowheads* point to GFP+/BrdU+ cells. *Arrows* point to GFP+/BrdU− cells. **e** Quantification of the mean percentages ± standard error of the mean of BrdU+/GFP+ cells of total GFP+ cells in the dorsal NT (about 10 nuclei were counted per hemi-NT; *P < 0.005). **f–i’** caAlk3 downregulates Foxd3 mRNA (**g**, **g’**, *arrow*) and prematurely upregulates Hairy1 to a level similar to that apparent in the floor plate (*FP*; **i**, **i’**, *arrow*) when compared to the corresponding contralateral sides or to control GFP-electroporated NTs that show a similar level of bilateral transcript expression (**f**, **f’**, **h**, **h’**). Bar for **a**, **b** = 50 μM; **c**, **d** = 40 μM; **c’**, **c”** **d’**, **d”** = 30 μM; **f**–**i’** = 40 μM

Next, we examined the effects of caAlk3 on NC EMT, G1/S transition, and gene expression. Electroporations were directed ventro-dorsally, transfecting either the dorsal quadrant of hemi-NTs or their dorsal domain on both sides of the midline. Consistent with our hypothesis, NC EMT was dramatically inhibited upon transfection of caAlk3 (N = 7) when compared to controls (N = 5, Fig. 4a, b), as was the extent of BrdU incorporation into the nuclei of transfected progenitors when challenged for a prolonged pulse of 2 h (N = 7 and 6, respectively, *P* < 0.005, Fig. 4c–e). In addition, by 12 h *Foxd3* mRNA was downregulated in the caAlk3-electroporated progenitors (N = 5, Fig. 4g, g’ arrow) compared to control GFP (N = 3, Fig. 4f, f’), confirming that NC production was inhibited. Reciprocally, expression of the RP marker *Hairy1* (Additional file 1: Figure S1K, L) was upregulated as early as 9 h post-transfection to levels similar to those apparent in the FP (N = 8, Fig. 4i, i’ arrow) whereas control GFP had no effect (N = 4, Fig. 4h, h’).

Whereas NC proliferation and Foxd3 expression are inhibited by high BMP signaling, our previous results showed that inhibiting endogenous BMP activity by noggin at the early NC stage also attenuates NC proliferation, Foxd3 expression, and NC EMT [16, 45, 46]. Here, we further confirm and extend these data by showing that treatment with noggin, Smad6 (a negative effector of BMP signaling [47]), or a dominant negative form of BMP receptor (dnBMPR) [48] all caused a significant reduction of BrdU incorporation in premigratory NC cells 16 h after electroporation when compared to control GFP-treated embryos (N = 4 embryos per treatment, *P* < 0.05, Additional file 4: Figure S4). Hence, both early inhibition as and high levels of BMP signaling compromise NC production. However, abrogation of endogenous BMP signaling did not promote a premature upregulation of *Hairy1* expression (N = 5, Additional file 4: Figure S4F), in contrast to the premature appearance of Hairy1 transcripts observed upon caAlk3 treatment. In addition, no *Hairy1* mRNA was apparent at the RP stage in embryos treated with either dnBMPR or noggin when compared to controls (N = 4 for each treatment, Additional file 5: Figure S5). Together, these results suggest that BMP signaling is both necessary as well as sufficient for expression of Hairy1 in RP and further indicate that development of RP properties requires high BMP signaling.

### Dynamics and function of Hes/Hairy1 in the dorsal NT

#### Hes1/Hairy1 activity is restricted to the RP stage

**Fig. 5:**
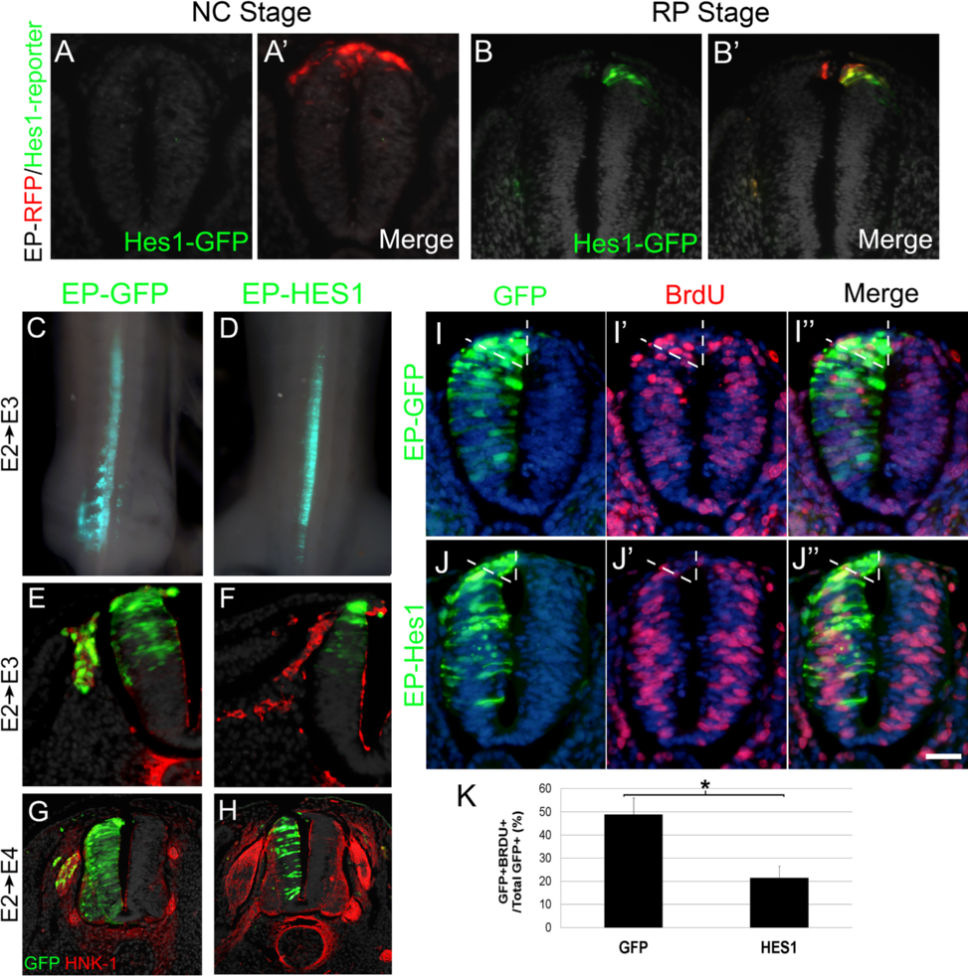
Misexpression of Hes1 at the neural crest (*NC*) stage inhibits G1/S transition and NC emigration. **a**–**b’** Co-electroporation of the dorsal neural tube (NT) at the NC (17 ss) or RP (40 ss) stages, respectively, with control RFP (*red*) and a Hes1 reporter-GFP. Ten hours later, Hes activity is apparent in RFP+ cells only at the roof plate (*RP*) stage (**b**, **b’**). **c–j** Hemi-NT electroporations of control GFP or Hes1 at the NC stage, analyzed 16 h (**i**, **j**), 24 h (**c**–**f**), or 48 h (**g**, **h**) after transfection. Dorsal views of whole embryos (**c**, **d**) and sections stained for the migrating NC marker HNK-1 (**e**–**h**) showing the absence of migrating Hes1+ cells in **d**, **f**, and **h** compared to GFP+ controls in **c**, **e**, and **g**. **i**, **j** A BrdU incorporation assay (1-h incubation) showing reduced incorporation in the dorsal NT of embryos electroporated with Hes1 compared to GFP controls. Nuclei are visualized with Hoechst (*blue*). *Dashed lines* delimit the analyzed dorsal NT domain. **k** Quantification of the mean percentages ± standard error of the mean of BrdU+ cells in the dorsal NT (*P < 0.01). Bar in **b**, **e**, **f** = 50 μM; **g**, **h** =70 μM; **a**, **i**–**j**” = 40 μM

*Hes1/Hairy1* is a candidate gene possibly involved in the transition between NC and RP stages because it is prematurely transcribed upon exposure of dorsal NT cells to high BMP signaling levels (Fig. 4i). Furthermore, *Hairy1* mRNA is expressed at the RP but not the NC stage (Additional file 1: Figure S1). To further evaluate its involvement in this transition, we implemented the mouse Hes1 promoter, homologous to chick Hairy1 [49], that drives expression of a reporter GFP cassette [50]. Co-electroporation of the Hes1/GFP promoter and control RFP at the NC stage (17–18 ss) showed no GFP signal in the RFP+ progenitors when examined 10 h later (N = 6, Fig. 5a, a’). In contrast, the RFP+ RP cells were GFP+ when co-electroporation was performed at 40 ss (N = 5, Fig. 5b, b’). Thus, consistent with its mRNA expression pattern, Hes1 is specifically active in the RP.

#### Hes1/Hairy1 inhibits NC delamination and cell-cycle progression

Next, we examined whether misexpression of Hes/Hairy1 at the NC stage may adversely affect NC behavior. We observed no delamination either 1 or 2 days after electroporation of Hes1, the mouse Hairy1 homologue (Fig. 5d, f, h), when compared to control GFP-electroporated embryos (Fig. 5c, e, g). Next, we found that Hes1 misexpression promotes premature cell-cycle exit in the electroporated cells, evidenced by the inhibition of BrdU incorporation when compared to GFP controls (N = 5 for each treatment, Fig. 5i–k, *P* < 0.01).

#### Hes1/Hairy1 attenuates BMP responsiveness in the dorsal NT and downregulates Alk3 mRNA expression

The preceding results indicate that Hes1 plays a role in ending NC production. This could be accounted for by Hes1 exerting a negative feedback on BMP signaling or on genes acting downstream of BMP. Such a Hes1-dependent loss of endogenous BMP activity in dorsal NT cells might explain the loss of responsiveness to BMP observed upon transition into the RP stage, which causes a concomitant exit from the cell cycle and the end of NC EMT.

**Fig. 6:**
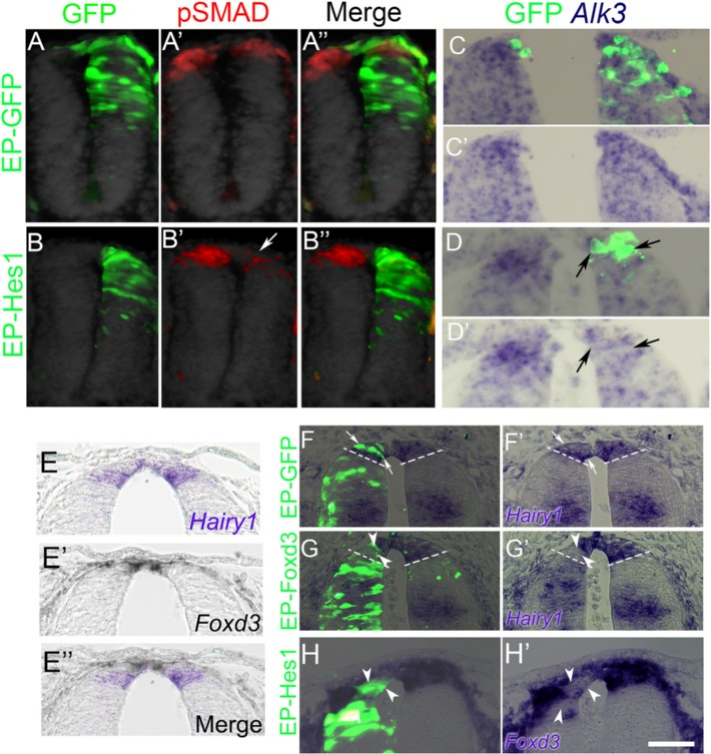
The relationship between Hes1/Hairy1, BMP signaling, and downstream *Foxd3* expression. **a**–**b”** Embryos were electroporated with either control GFP (**a**–**a”**) or Hes1 (**b**–**b”**) at the neural crest (NC) stage and analyzed 20 h later for pSMAD immunostaining (*red*). Note the decreased pSMAD signal in the treated side at **b’** (*arrow*) compared to the untransfected side and to control GFP. **c**–**d’** Embryos were electroporated with either control GFP (**c**, **c’**) or Hes1/GFP (**d**, **d’**) at the NC stage and analyzed 15 h later for *Alk3* mRNA expression. Note the reduction in the Alk3 signal in the transfected (*green*) side of the Hes-treated hemi-neural tube (NT) (*white arrow*). **e**–**e”** Mutually exclusive spatial expression domains of *Foxd3* and *Hairy1*. In situ hybridization on adjacent sections of the same embryo performed at 35 ss. Note that at this particular stage, *Hairy1* expression (*blue*) is ventral to the *Foxd3*+ (*black*) domain. **f**, **g’** Foxd3 and Hairy1 inhibit each other’s expression. Electroporation of Foxd3 at E2.5 cell autonomously inhibits *Hairy1* expression in the dorsal NT 16 h later (**g**, **g’**; *arrowheads*), compared to the contralateral side and to GFP-electroporated controls (**f**, **f’**; *arrows*). **h**, **h’** Electroporation of Hes1 at the NC stage inhibits *Foxd3* transcription compared to the contralateral side. *Arrowheads* point to GFP+/*Foxd3*− cells. For control see Fig. 4f. Bar for **a**–**b”**, **f**–**g’** = 60 μM; **c**–**e”**, **h**, **h’** = 50 μM

To examine this possibility, Hes1 was electroporated at the NC stage, and pSMAD expression analyzed 20 h later. In control GFP-transfected tubes, pSMAD staining was similarly expressed on both sides of the dorsal NT (Fig. 6a–a”). In contrast, NTs transfected with Hes1/Hairy1 displayed a significant reduction in pSMAD staining in the transfected hemi-NTs (Fig. 6b–b”, arrow in b’).

Next, we asked whether the Hes1-dependent loss of BMP activity can be explained by a downregulation of Alk3 receptors, whose expression is normally lost in the transition between NC and RP stages (see Additional file 2: Figure S2). Fifteen hours after the co-electroporation of Hes1 and GFP-encoding plasmids at the NC stage, we observed a premature reduction of *Alk3* mRNA signal in the transfected hemi-NTs (N = 9, Fig. 6d, d’) when compared to control GFP alone (N = 6, Fig. 6c, c’).

Thus, the onset of Hairy1 production in the nascent RP might be sufficient to inhibit both BMP receptor expression and BMP responsiveness, consequently abrogating BMP-dependent G1-S transition and cell delamination.

#### A mutual cross-inhibition between Foxd3 and Hairy1

The transcription factor Foxd3 is a BMP-dependent gene expressed from early stages onwards in the dorsal NT [4, 13, 16, 51]. Consistently, *Foxd3* is transcribed at the NC but not definitive RP stages (Additional file 1: Figure S1G, H) [12, 13]. Conversely, expression of *Hes1*/*Hairy1* is largely reciprocal to that of *Foxd3* (Additional file 1: Figure S1K, L and Fig. 5). To further examine their differential expression, we performed in situ hybridization on embryos aged 33–36 ss (HH17–18), an intermediate stage corresponding to the dorsal restriction of the *Foxd3*-positive domain [12] and the onset of *Hairy1* transcription (e.g., the transition between NC and RP stages). In these embryos, *Foxd3* and *Hairy1* were not co-expressed in the same cells. Whereas *Foxd3* mRNA was already restricted to a narrow strip of progenitors located in the dorsal midline, *Hairy1* expression was apparent immediately ventral to the *Foxd3*-positive domain, presumably corresponding to prospective RP progenitors (Fig. 6e, e’). These results further strengthen the dynamic behavior of dorsal NT precursors where RP progenitors are situated ventral to the premigratory NC prior to arriving at their definitive position [12].

Based on their reciprocal expression patterns, we hypothesize that Foxd3 and Hes1/Hairy1 may stand in a mutually repressive interaction. Misexpression of Foxd3 close to the end of the NC stage inhibited in a cell-autonomous manner the transcription of *Hairy1* when analyzed at the RP stage (N = 5, arrowheads in Fig. 6g, g’) in comparison to GFP controls (N = 5, arrows in Fig. 6f, f’). In addition, misexpression of Hes1 at the NC stage repressed *Foxd3* mRNA in the dorsal NT when compared to the untreated side and to control GFP (N = 6, arrowheads in Fig. 6h, h’ and see Fig. 4f, f’ for control GFP).

These results suggest that Foxd3 and Hairy1 negatively regulate each other, thus maintaining both spatial and temporal separation between NC and RP properties in the dorsal neuroepithelium.

## Discussion

During the development of the CNS, neuroepithelial cells gain distinct identities and give rise to numerous cell types. In the region of the NT destined to become the spinal cord, the acquisition of distinct fates is coordinated by the RP and FP, organizing centers secreting diffusible instructive signals. But how do the organizing centers themselves form and gain their proper fates? While significant work has been invested in understanding FP development [52], very little is known about the formation of the RP. This is further complicated by the highly dynamic behavior of this region, which is first populated by NC progenitors and only after their exit from the NT becomes the RP, a definitive group of CNS cells.

**Fig. 7:**
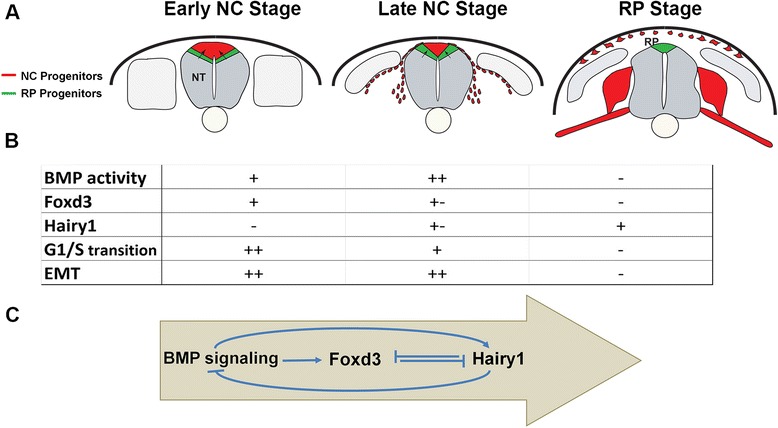
The dynamics of dorsal neural tube (*NT*) development: transition from neural crest (*NC*) to definitive roof plate (*RP*). **a** Before the onset of NC migration (*left panel*), presumptive RP progenitors (*green*) are located ventral to the premigratory NC (*red*). They progressively reposition dorsally upon the onset and progression of NC emigration (*middle panel*, red cells outside the NT are migrating NC) until reaching their definitive localization at the dorsal midline of the central nervous system primordium (*right panel*). *Arrows* depict the ventral to dorsal direction of cellular relocation. For details see [12, 13]. **b**, **c** At the early NC stage, BMP signaling becomes active in the dorsal NT (+), inducing a series of BMP-dependent genes such as *Foxd3*, and promoting cell-cycle progression and subsequent NC epithelial-to-mesenchymal transitions (*EMT*) and delamination. As time goes on, NC cells leave the dorsal NT while RP progenitors are exposed to BMP signaling for a longer duration, perhaps interpreted as a higher signaling level (++). This induces initial *Hairy1* synthesis ventral to the progressively narrowing domain of *Foxd3* (see also [12]). At the RP stage, Hairy1-expressing cells reach the dorsal midline domain and Hairy1 inhibits expression of Alk3 receptors and further responsiveness of RP cells to BMP signaling (−), which results in the inhibition of *Foxd3* transcription, arrest of cell-cycle progression, and the end of cellular EMT, altogether contributing to the consolidation of the definitive RP. Given that misexpression of Foxd3 close to the RP stage inhibits *Hairy1*, these cross-repressive interactions may account for the spatial and temporal separation of NC and RP lineages. The *arrow* in **c** depicts a time sequence

Here, we have begun to unravel the mechanism that accounts for the transition between NC and RP stages. First, we have identified a number of cellular and molecular traits that characterize each stage. Second, we report that although initially responsive to BMP, RP progenitors lose competence to respond to the ligand upon transition to their definitive location. In parallel, RP cells upregulate *Hairy1*, which is likely to confer the observed insensitivity to BMP signaling, despite the fact that they continuously synthesize the ligand. Consequently, NC production and emigration end, presumably due to a cell-cycle arrest induced by Hairy1 via downregulation of BMP receptors and consequent BMP signaling. In addition, *Foxd3* and *Hairy1* not only have non-overlapping temporal expression patterns, but also inhibit each other’s transcription. Together, this constitutes a negative regulatory loop at the level of, and downstream from, BMP signaling that controls the sequential production of NC followed by RP (Fig. 7).

Classically, the term RP is generically used to define the dorsal domain of the NT, in particular with regard to its morphogen-secreting capacity and function in patterning of dorsal interneurons [14, 22, 39]. However, compounds that are usually considered as RP markers—such as morphogens like BMPs and Wnts, and transcription factors like MafB and Lmx1a/b—are produced throughout dorsal NT development, encompassing the early NC period [4, 5]. Clearly, the dorsal NT differs significantly between the stages both in terms of cell fates and cellular behaviors, demonstrating the need for a more exact definition.

Our previous experiments [12, 13] showed that progenitors of the definitive RP are initially located ventral to the prospective NC. Initially, these cells are molecularly indistinguishable from presumptive NC because they also express *Foxd3*, as evidenced by the use of a Foxd3 reporter [13], and are still responsive to BMP, as revealed here by lineage analysis with a BRE-GFP reporter. Upon NC emigration, prospective RP cells relocate dorsally toward their definitive midline position, and during this time they become refractory to BMP, downregulate *Alk3*, and cease to express *Foxd3* and the direct BMP target genes *Id2/3*. The initiation of *Hairy1* expression must be associated with these events, because it is initially evident in a band of cells localized ventrally to the *Foxd3*+ domain; later, when BMP responsiveness and *Foxd3* transcription are lost, Hairy1 expression and activity are evident in the most dorsal NT domain. This correlates in time with the completion of NC emigration.

This view would suggest that the functions of BMP and Wnt signaling differ between the NC and RP stages. Studies so far have addressed the role of these signaling molecules on various aspects of interneuron development yet misexpressed the ligands starting at the NC stages and analyzed the outcome 2 days later, thus encompassing both phases [8, 18, 53]. Clearly, limiting the loss of either BMP or Wnt function to the NC phase inhibits G1/S transition and the consequent EMT of these precursors by activating a molecular network that includes N-cadherin processing and Rho-GTPase activity [9, 17, 38, 54]. However, it remains unclear whether such an early and time-limited inhibition suffices for preventing or altering interneuron development, and whether the latter cells require a continuous supply of the factors or, alternatively, whether a late supply by the definitive RP suffices given that interneuron progenitors still exhibit BMP responsiveness at this stage (Fig. 2). Another possibility would be that signals emanating from the young dorsal NT are important for interneuron specification and then differentiation [8, 18], whereas those from the definitive RP are necessary for axonal outgrowth and/or guidance. Taken together, we sustain that the use of the term RP to describe the structure emerging upon NT closure is inappropriate and propose instead to use the term RP only when NC delamination has ended, and the full segregation between CNS and PNS lineages is evident.

Members of the Hes/Hairy family of transcriptional repressors were found to be constitutively expressed in neural boundary domains, like the RP and FP of the spinal cord [22]. Persistent and high Hes1 expression levels repress both proneural gene transcription and cell proliferation in boundary regions within the nervous system [22, 55], whereas in the absence of Hes, *Mash1* and other proneural genes are ectopically expressed in these domains [22, 56]. In addition, an oscillatory expression of Hes genes has been documented in developing neuroblasts. This oscillatory behavior was critical to maintain progenitor cells in an undifferentiated state while inhibiting neuronal differentiation [57]. Furthermore, Hes/Hairy genes were found to play a role in regulating cell-cycle progression [58]. Whereas low levels of Hes1 promote cell proliferation by downregulating p21 and p27 [59], persistent and high levels of Hes1 were shown to inhibit the cell cycle [22, 60]. Consistently, as shown here, the advent of the RP is associated with cell-cycle exit and the onset of *Hairy1* transcription. Moreover, Hes misexpression at the NC stage inhibited G1/S transition and NC production, suggesting that Hairy1 is functionally involved in ending NC production and RP formation.

An open question is what are the mechanisms that trigger Hes1/Hairy1 expression at the RP stage. Because both NC and RP precursors lie within a domain of high BMP signaling that expresses *Msx1/2* [18], we speculate that it is the comparably extended exposure of the latter to BMP that induces RP properties including *Hairy1*; this might occur by translating the longer duration of signal into a higher level of activity [44, 61] as suggested by our caAlk3 experiments. In addition, given that Foxd3 represses transcription of *Hairy1* (our data) and suppresses interneuron development [62], it is logical to assume that *Hairy1* transcription can only be stabilized in RP cells when *Foxd3* itself is repressed or when *Foxd3*+ NC progenitors exit the NT. Our data moreover suggest that Hairy1 itself contributes to the inhibition of *Foxd3* mRNA, either directly or indirectly by abrogating the responsiveness of dorsal NT cells to BMP signaling, the latter being a positive regulator of *Foxd3* transcription [4, 16, 51]. Indeed, high levels of BMP activity (and/or longer signal duration) might be sufficient for inducing *Hairy1* and repressing *Foxd3* transcription in the dorsal NT, thus generating a feedback network of transcriptional interactions that consolidate RP formation (see Fig. 7).

In addition to expressing *Hes/Hairy*, FP and RP share similar roles because these boundary domains act as organizing centers that secrete morphogens to pattern neuronal differentiation in adjacent cells. Previously, high concentrations of the Shh morphogen, which is secreted by the notochord, were shown to be needed in both mouse and avian embryos to specify the ventral midline domain as FP. Later, to complete FP differentiation, these cells become refractory to the ligand [24, 52]. In addition, *Foxj1* is upregulated in FP cells in association with the reduction in Shh responsiveness, and Foxj1 alters the sensitivity of cells to Shh signaling, presumably by inducing long, motile cilia [24]. By analogy to the FP, we show here that upon NT closure and during the NC stage, dorsal progenitors respond to BMP, but while transiting to the dorsal midline they downregulate Alk3 receptors, stop responding to BMP, and similarly upregulate *Foxj1*, regaining apically organized Arl13b + cilia and apico-basal polarity. Thus, in spite of the delayed development of the RP when compared to the FP [10], there are notable similarities between the specification of both ventral and dorsal organizing centers. Further investigation is needed to substantiate the mechanistic similarities between FP and RP development by examining, for instance, whether Foxj1 in the RP similarly alters BMP responsiveness and cilia morphology and function.

## Conclusions

By initially characterizing a set of positive and negative activities involving regulated BMP signaling and Hes/Foxd3 interactions, our results provide novel insights into the dynamic events leading to the transition from the NC to the RP phase of NT development. Four main processes are noteworthy: first, our finding that RP progenitors initially respond to BMP yet lose competence upon relocation to their definitive dorsal midline position in the NT, where they finally consolidate their identity; second, that BMP signaling induces Hes transcription, which in turn downregulates BMP responsiveness, likely through modulation of Alk3 receptor transcription; third, that downstream of BMP, a cross-repressive interaction between Foxd3 (an NC marker) and Hairy1 (an RP marker) accounts for the temporal and spatial segregation of both lineages; and fourth, that in spite of being refractory, the definitive RP continues producing BMP, which is likely to act upon dorsal interneurons. The precise time-dependent activities of BMP emanating from the early (NC stage) versus late (RP stage) dorsal NT remain to be defined. These multiple roles of BMP signaling indicate that its function is context dependent and dictated by the regulatory state and competence of the target cells. We also notice that RP ontogeny bears significant resemblance to the development of the FP, initiated by Shh signaling in the ventral NT, both in terms of signal duration/intensity followed by refractoriness. Future research should focus on unveiling additional genes and interactions that comprise the differential molecular networks underlying the sequential functions of BMP on NC, RP, and interneuron development.

## Methods

### Embryos

Chick (*Gallus gallus*) and quail (*Coturnix japonica*) eggs were obtained from commercial sources (Moshav Orot and Moshav Mata, respectively). Experiments were conducted at the flank level of the axis (20–25 ss).

### Expression vectors and electroporation

Expression vectors were: pCAGGS-EGFP, pCAGGS-RFP [12], pBI-EGFP, Noggin [9], pCAB-Smad6, pCAB-dnBMPR [48], pBI-mHes1 (subcloned from [50]), pBI-cFoxd3 [43, 62], and pBI-caBMPR1a/Alk3 (from D. Schulte and subcloned into pBI). To trace Hairy1 activity, a 2.5 kb mouse Hes1 promoter driving expression of a GFP reporter (pHes1-promoter-GFP) was used (N. Jing [50]). The specificity of the Hes1 reporter was previously assessed by monitoring changes to Notch signaling [33]. To monitor BMP signaling, we implemented a BRE that drives expression of a GFP reporter plasmid. BRE-GFP contains two copies of two distinct and conserved elements of the binding sites for Smad4 upstream of a minimal thymidine kinase promoter (E. Marti; [8]). Specificity of the BMP reporter was previously verified by co-electroporation with pEFBOS-mBMP4 [33].

Both the pHes1 promoter and BRE-GFP plasmids were electroporated along with a control RFP-encoding vector, to monitor electroporation efficiency.

DNA (2–5 mg/ml) was microinjected into the lumen of the NT at the trunk level of the axis at specific stages as detailed for each experiment. For hemi-NT electroporations, 5 mm tungsten electrodes were placed on either side of the embryo. For discrete electroporations into the dorsal NT, a 5 mm tungsten electrode was inserted under the blastoderm and a fine, 1–2 mm long electrode placed over the dorsal NT. A square wave electroporator (BTX, San Diego, CA, USA) was used to deliver one to three pulses of current at 10–20 V for 10 ms.

### Immunohistochemistry and in situ hybridization

Antibodies against HNK1 (CD57, BD Biosciences, San Jose, CA, USA Cat#559048, 1:500), Arl13b (from Tamara Caspary, 1:1000), ZO-1 (Thermo Fisher Scientific, Waltham, MA USA, cat#402200, 1:100), N-cadherin (R&D Systems, Minneapolis, MN, USA., cat#BTA7, 5 μg/ml), laminin (Sigma-Aldrich, St. St. Louis, MO, USA cat#L9393,1:100), BrdU (G3G4, Developmental Studies Hybridoma bank, Iowa City, Iowa, USA 1:100), and phosphorylated Smad 1-5-8 (pSMAD, from Ed Laufer, 1:1000) were used as previously described [9]. Cell nuclei were visualized with Hoechst. In situ hybridization was performed on paraffin sections as described [25]. The following probes were employed: *BMP4* [16], *Foxd3* [62], *Bmpr1a*/*Alk3*, *Bmpr1b/Alk6* [63], *Hairy1* [49], *Id2*, *Id3* [33], *Foxj1* [64], *MafB* [65], *Wnt1* [4], and *Gdf7* (from A. Graham).

### Data analysis and statistics

The dorsal NT domain at NC and RP stages was analyzed. At the NC stage, the expression domain of *Foxd3* generally contains 8–14 (9.57 ± 1.18) nuclei, and the expression domain of *MafB* at the RP stage contains 10–15 (13.5 ± 0.94) nuclei per hemi-NT. Therefore, for cell counts and domain definition at either stage, the most dorsal 8–10 nuclei per hemi-NT were considered.

Five to twelve embryos were analyzed per experimental treatment. For BrdU incorporation measurements, cells in 5–20 sections per embryo were counted and expressed as percentage of BrdU+/total GFP+ cells in the dorsal NT.

Images were photographed using a DP70 (Olympus, Japan) cooled charge-coupled device digital camera mounted on a BX51 microscope (Olympus, Japan). Confocal sections of whole-mount preparations encompassing their entire thickness were photographed using a Nikon Eclipse 90i microscope with a Plan Apo 10×/0.45 dry objective (Nikon, Japan) and a D-Eclipse c1 confocal system (Nikon, Japan) at 2.7 μm increments through the z-axis. Images were z-stacked with EZ-C1 3.90 FreeViewer software. For figure preparation, images were exported into Photoshop CS6 (Adobe). If necessary, the levels of brightness and contrast were adjusted to the entire image and images were cropped without color correction adjustments or γ adjustments. Final figures were prepared using Photoshop CS6.

Data were subjected to statistical analysis using the nonparametric Mann-Whitney and Kruskal-Wallis tests. All tests applied were two-tailed and a *P*-value ≤ 0.05 was considered significant.

## Additional files

Additional file 1: Figure S1. Stage-specific expression of selected dorsal NT markers. (A–H) In situ hybridization for *Id2* (A, B), *Id3* (C, D), *LTK* (E, F), and *Foxd3* (G, H) at NC and RP stages. Note selective expression at the NC stage and no expression in the definitive RP. Among additional expression patterns, both *Id2/3* are transcribed ventrally to the RP, corresponding to dorsal interneurons (B, D). (I–L) *Foxj1* and *Hairy1* are transcribed in the definitive RP (J, L) but not the dorsal NT at the NC stage (I, K). Note that both are expressed in the floor plate. (M–R) *Gdf7*, *Wnt1*, and *MafB* are expressed at both stages in the dorsal NT. Bar for A ,C ,E ,G ,I ,K ,M ,O , Q = 40 μM; B, D, F, H, J, L, N, P, R = 70 μM. (JPG 1246 kb) Additional file 2: Figure S2. Expression of BMP receptors in the NT. (A–E) In situ hybridization for *Bmpr1a/Alk3* (A–C) and *Bmpr1b/Alk6* (D, E) at NC (A, B, D) and RP stages (C, E). A and D depict an early NC stage opposite epithelial somites, and B illustrates an advanced migratory stage. Dashed lines in C and E delimit the definitive RP. Bar for A, B, D = 60 μM; C, E = 100 μM. (JPG 186 kb) Additional file 3: Figure S3. caBMPR1A/caAlk3 stimulates ectopic pSMAD activity. (A–A”) Electroporation of control GFP (PBI-GFP) or caBMPR1a-GFP-PBI (B–B”) followed by pSMAD immunostaining. Note in A–A” that the pSMAD signal is restricted to the dorsal NT even if the transfection attains half of the NT length. In contrast, caBMPR1a/Alk3 induces ectopic pSMAD activity in transfected cells throughout the electroporated domain (B–B”). Bar = 50 μM. (JPG 516 kb) Additional file 4: Figure S4. Inhibition of BMP signaling abrogates G1/S transition in the dorsal NT but does not stimulate premature *Hairy1* expression. (A–D) Transverse sections showing the NT following a 1 h pulse of BrdU. Embryos were sacrificed 16 h after electroporation of either control GFP (A–A”’), Noggin (B–B”’), Smad6 (C–C”’), or dnBMPR (D–D”’). Arrowheads point to GFP+/BrdU+ cells. Arrows point to GFP+/BrdU– cells. (E) Quantification of the mean percentages ± standard error of the mean of BrdU+/GFP+ cells of total GFP+ cells in the dorsal NT (about 10 nuclei were counted per hemi-NT, N = 4 embryos for each treatment; **P* < 0.05). Nuclei are visualized with Hoechst (blue). (F, F’) Misexpression of noggin-GFP (green) has no effect on the expression of Hairy transcripts. F’ is an overlay of noggin-GFP and *Hairy1* in situ hybridization. Bar = 40 μM. (JPG 700 kb) Additional file 5: Figure S5. Inhibition of BMP signaling prevents *Hairy1* expression in the dorsal midline region of the NT at the RP stage. Electroporations at the 10–15 ss of control GFP (A–A”), dnBMPR (B–B”), or noggin (C–C”) followed by fixation at 45 ss. Note *Hairy1* expression in the GFP+ RP of the control NT and the presence of many GFP-labeled, NC-derived cells in the DRG. In contrast, no *Hairy1* signal is apparent in the hemi-RP misexpressing dnBMPR-GFP (asterisk in B’), or in the entire RP that received noggin/GFP (asterisk in C’). Arrows in A’, B’, and C’ point to the RP. As expected from the known effects of BMP signaling on NC delamination, few or no labeled NC-derived cells were observed in peripheral targets of these embryos. *DRG* dorsal root ganglion, *FP* floor plate, *NT* neural tube. Bar = 60 μM. (JPG 858 kb)

## Abbreviations

bHLH: basic helix-loop-helix
BMP: bone morphogenetic protein
BMPR: bone morphogenetic protein receptor
BrdU: bromodeoxyuridine
BRE: BMP-responsive element
ca: constitutively active
CNS: central nervous system
DRG: dorsal root ganglion
E: embryonic day
EMT: epithelial-to-mesenchymal transition
EP: electroporation
FP: floor plate
GFP: green fluorescent protein
HH: Hamburger and Hamilton stage
LTK: leukocyte tyrosine kinase
NC: neural crest
NT: neural tube
PNS: peripheral nervous system
RFP: red fluorescent protein
RP: roof plate
Shh: Sonic hedgehog
ss: somite stage
