# Supplementary figures and images for: Dynamics of BMP and Hes1/Hairy1 signaling in the dorsal neural tube underlies the transition from neural crest to definitive roof plate
Source: BMC Biol. 2016 Mar 24;14:23. doi: 10.1186/s12915-016-0245-6 (PMC4806459; doi:10.1186/s12915-016-0245-6)

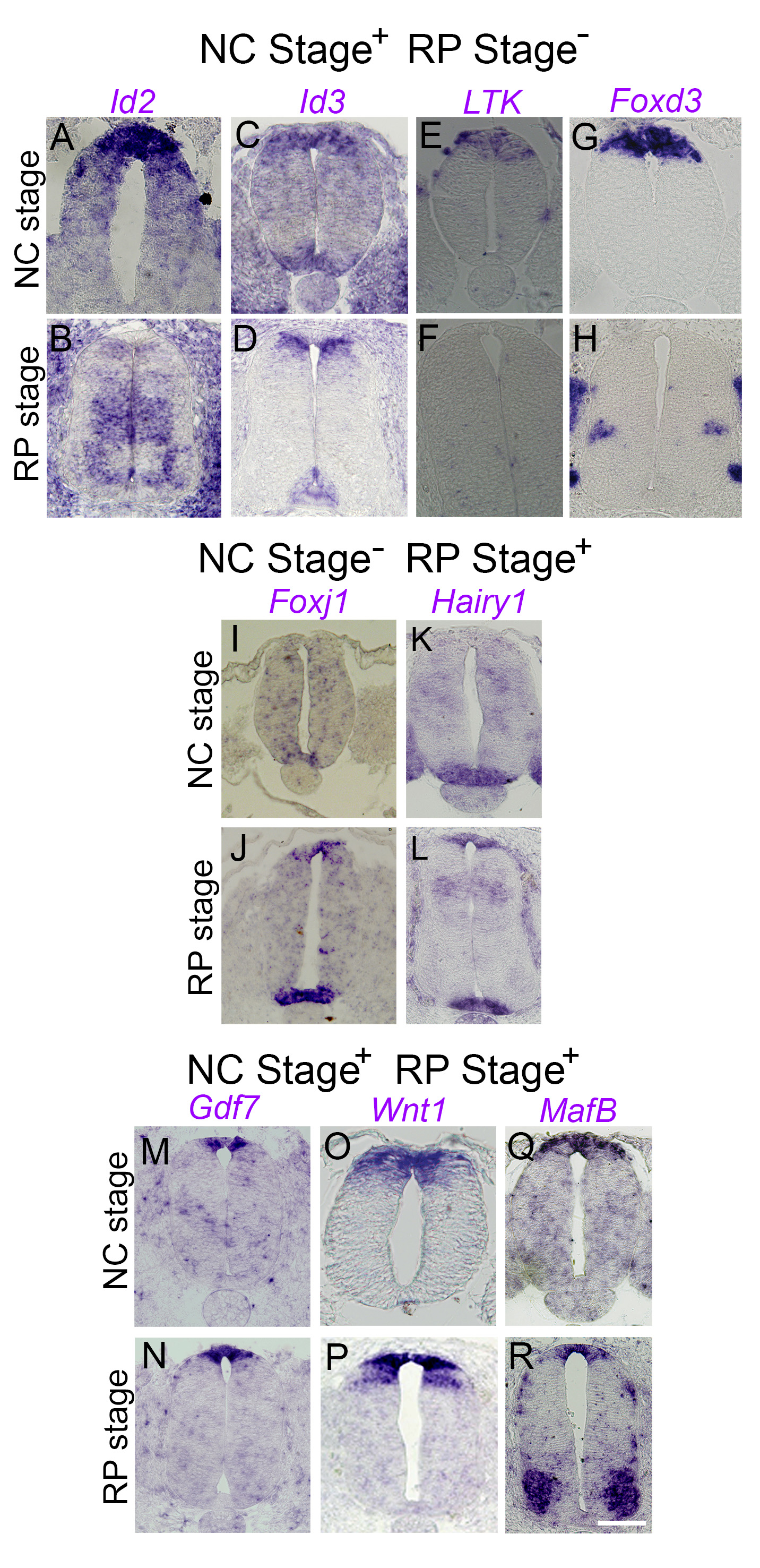

Supplement: Additional file 1: Figure S1. — Stage-specific expression of selected dorsal NT markers. (A–H) In situ hybridization for Id2 (A, B), Id3 (C, D), LTK (E, F), and Foxd3 (G, H) at NC and RP stages. Note selective expression at the NC stage and no expression in the definitive RP. Among additional expression patterns, both Id2/3 are transcribed ventrally to the RP, corresponding to dorsal interneurons (B, D). (I–L) Foxj1 and Hairy1 are transcribed in the definitive RP (J, L) but not the dorsal NT at the NC stage (I, K). Note that both are expressed in the floor plate. (M–R) Gdf7, Wnt1, and MafB are expressed at both stages in the dorsal NT. Bar for A ,C ,E ,G ,I ,K ,M ,O , Q = 40 μM; B, D, F, H, J, L, N, P, R = 70 μM. (JPG 1246 kb) [file 12915_2016_245_MOESM1_ESM.jpg]

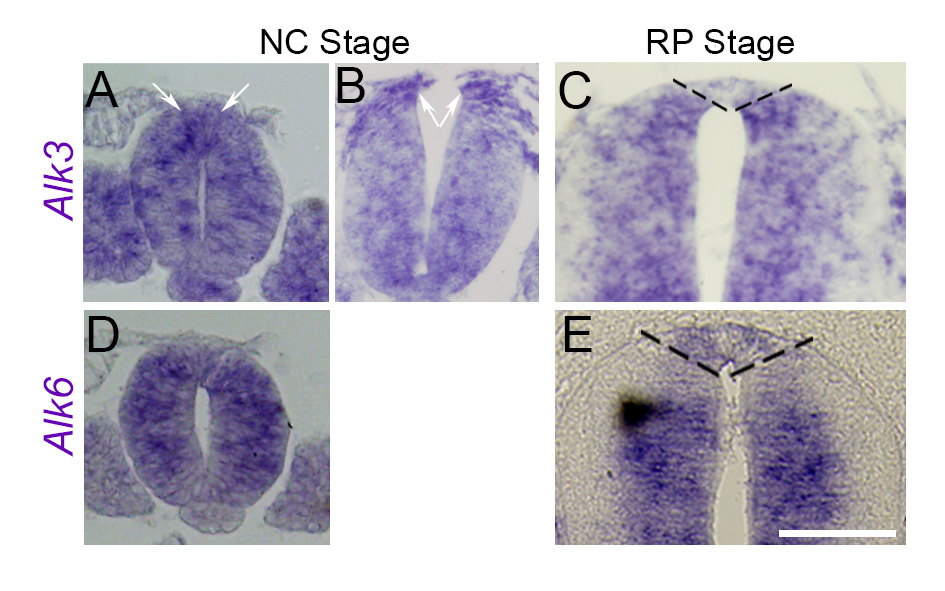

Supplement: Additional file 2: Figure S2. — Expression of BMP receptors in the NT. (A–E) In situ hybridization for Bmpr1a/Alk3 (A–C) and Bmpr1b/Alk6 (D, E) at NC (A, B, D) and RP stages (C, E). A and D depict an early NC stage opposite epithelial somites, and B illustrates an advanced migratory stage. Dashed lines in C and E delimit the definitive RP. Bar for A, B, D = 60 μM; C, E = 100 μM. (JPG 186 kb) [file 12915_2016_245_MOESM2_ESM.jpg]

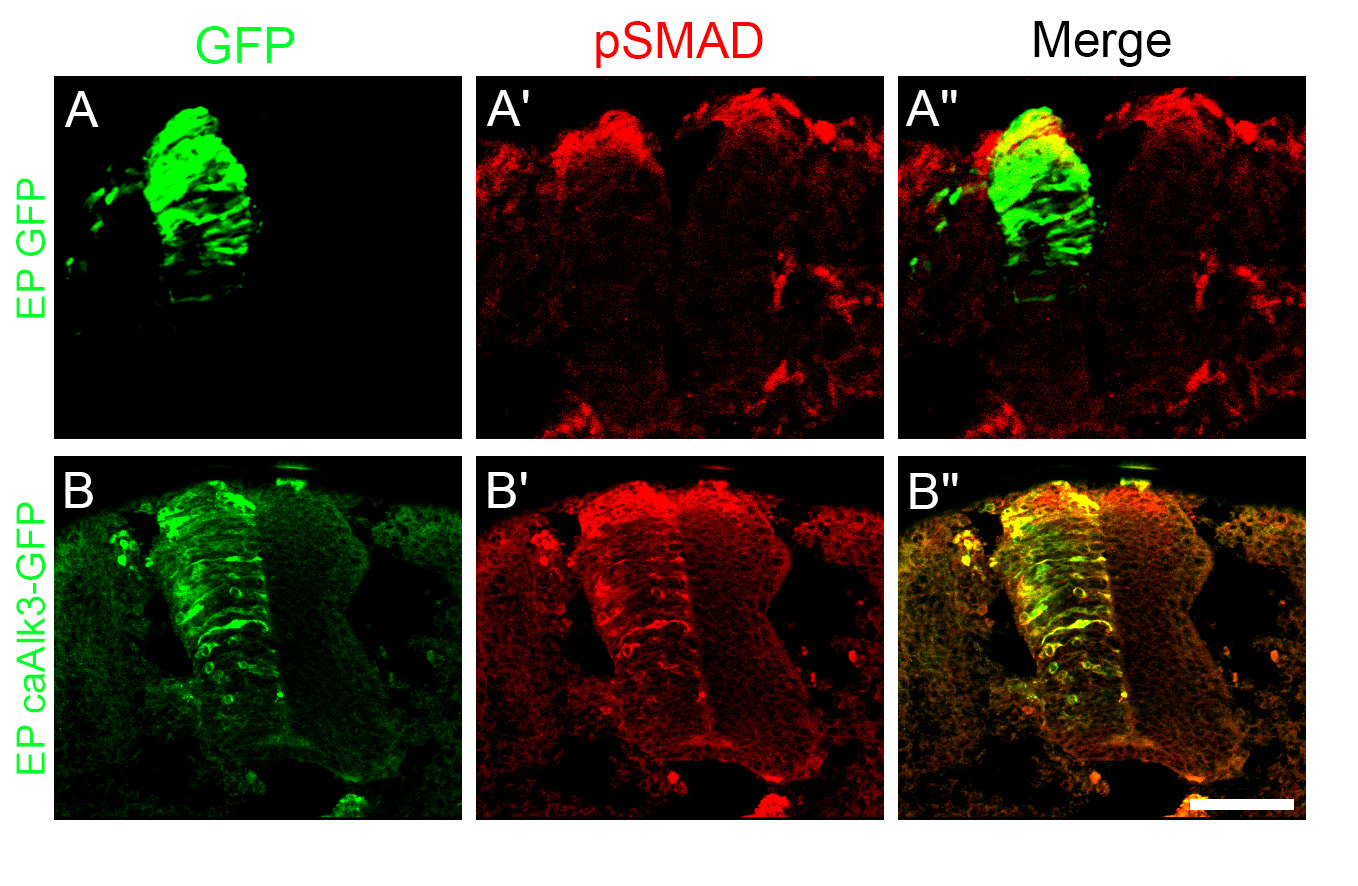

Supplement: Additional file 3: Figure S3. — caBMPR1A/caAlk3 stimulates ectopic pSMAD activity. (A–A”) Electroporation of control GFP (PBI-GFP) or caBMPR1a-GFP-PBI (B–B”) followed by pSMAD immunostaining. Note in A–A” that the pSMAD signal is restricted to the dorsal NT even if the transfection attains half of the NT length. In contrast, caBMPR1a/Alk3 induces ectopic pSMAD activity in transfected cells throughout the electroporated domain (B–B”). Bar = 50 μM. (JPG 516 kb) [file 12915_2016_245_MOESM3_ESM.jpg]

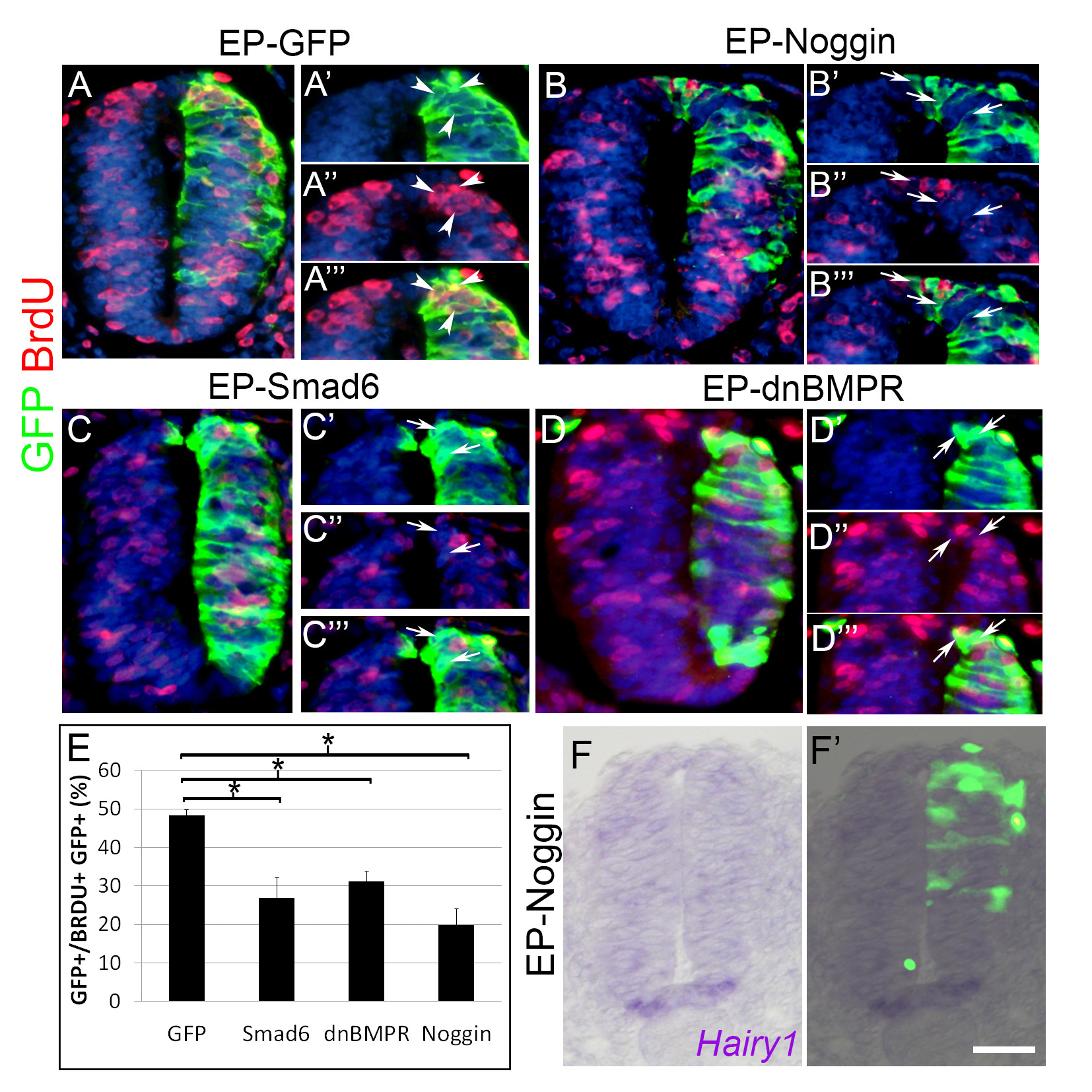

Supplement: Additional file 4: Figure S4. — Inhibition of BMP signaling abrogates G1/S transition in the dorsal NT but does not stimulate premature Hairy1 expression. (A–D) Transverse sections showing the NT following a 1 h pulse of BrdU. Embryos were sacrificed 16 h after electroporation of either control GFP (A–A”’), Noggin (B–B”’), Smad6 (C–C”’), or dnBMPR (D–D”’). Arrowheads point to GFP+/BrdU+ cells. Arrows point to GFP+/BrdU– cells. (E) Quantification of the mean percentages ± standard error of the mean of BrdU+/GFP+ cells of total GFP+ cells in the dorsal NT (about 10 nuclei were counted per hemi-NT, N = 4 embryos for each treatment; *P < 0.05). Nuclei are visualized with Hoechst (blue). (F, F’) Misexpression of noggin-GFP (green) has no effect on the expression of Hairy transcripts. F’ is an overlay of noggin-GFP and Hairy1 in situ hybridization. Bar = 40 μM. (JPG 700 kb) [file 12915_2016_245_MOESM4_ESM.jpg]

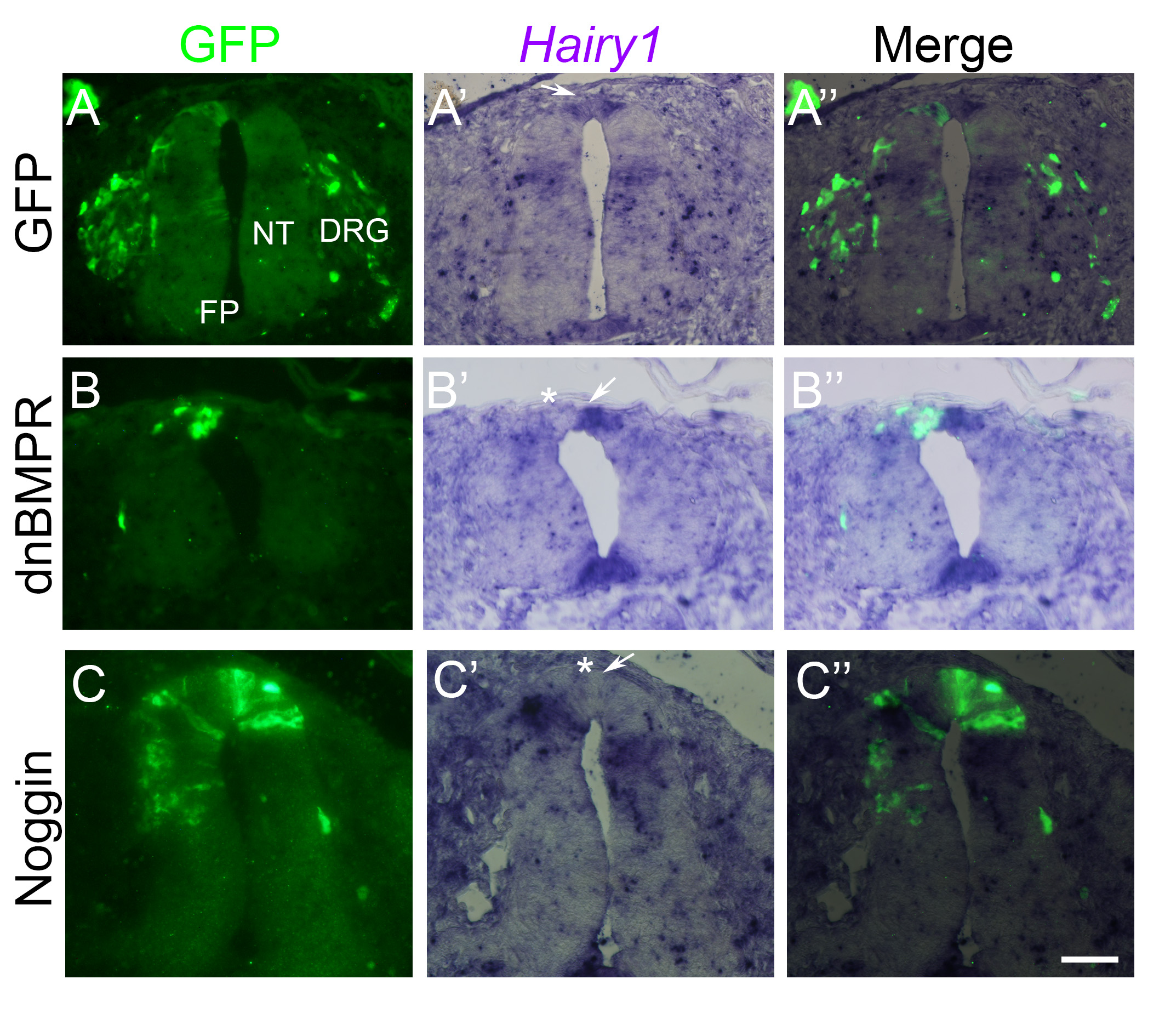

Supplement: Additional file 5: Figure S5. — Inhibition of BMP signaling prevents Hairy1 expression in the dorsal midline region of the NT at the RP stage. Electroporations at the 10–15 ss of control GFP (A–A”), dnBMPR (B–B”), or noggin (C–C”) followed by fixation at 45 ss. Note Hairy1 expression in the GFP+ RP of the control NT and the presence of many GFP-labeled, NC-derived cells in the DRG. In contrast, no Hairy1 signal is apparent in the hemi-RP misexpressing dnBMPR-GFP (asterisk in B’), or in the entire RP that received noggin/GFP (asterisk in C’). Arrows in A’, B’, and C’ point to the RP. As expected from the known effects of BMP signaling on NC delamination, few or no labeled NC-derived cells were observed in peripheral targets of these embryos. DRG dorsal root ganglion, FP floor plate, NT neural tube. Bar = 60 μM. (JPG 858 kb) [file 12915_2016_245_MOESM5_ESM.jpg]
